# Supplementary material for: Nonverbal leadership emergence in walking groups
Source: Sci Rep. 2020 Nov 3;10:18948. doi: 10.1038/s41598-020-75551-2 (PMC7609592; doi:10.1038/s41598-020-75551-2)
Supplement: Supplementary file 1 — Supplementary material 1 [file 41598_2020_75551_MOESM1_ESM.pdf]

Nonverbal leadership emergence in walking groups
# - Supplementary Information -

Maria Lombardi[1,2], William H. Warren[3], Mario di Bernardo[1,2]

[1] Department of Engineering Mathematics, University of Bristol, Bristol, UK

[2] Department of Electrical Engineering and Information Technology, University of Naples Federico II, Naples, Italy

[3] Department of Cognitive, Linguistic and Psychological Sciences, Brown University, Providence, RI 02912, USA

Contents

Supplementary tables **1**

Supplementary figures **4**

Supplementary tables

Details about each trial are reported in Table 1 and 2 respectively for Group 1 and Group 2. A total of 3 trials out of 54 were discarded in the preprocessing analysis for data acquisition issues.

**Trial**	**Condition**	**IPD** [m]	**Group**	**Pedestrian** [FL, FR, BL, BR]
1	direction (left, left)	4	1	[P1.1, P1.4, P1.2, P1.3]
2	direction (left, right)	2	1	[P1.2, P1.3, P1.1, P1.4]
3	direction (left, right)	4	1	[P1.4, P1.1, P1.3, P1.2]
4	direction (right, left)	2	1	[P1.1, P1.2, P1.4, P1.3]
5	direction (left, left)	2	1	[P1.3, P1.4, P1.2, P1.1]
6	direction (right, right)	1	1	[P1.4, P1.2, P1.3, P1.1]
7	direction (right, right)	2	1	[P1.1, P1.2, P1.3, P1.4]
8	direction (left, left)	1	1	[P1.1, P1.4, P1.3, P1.2]
9	direction (right, left)	4	1	[P1.2, P1.3, P1.4, P1.1]
10	direction (right, right)	4	1	[P1.1, P1.4, P1.3, P1.2]
11	direction (right, left)	1	1	[P1.4, P1.1, P1.3, P1.2]
12	control	2	1	[P1.2, P1.3, P1.4, P1.1]
13	control	4	1	[P1.3, P1.1, P1.4, P1.2]
14	speed (fast, slow)	2	1	[P1.4, P1.2, P1.1, P1.3]
15	speed (slow, slow)	2	1	[P1.3, P1.2, P1.1, P1.4]
16	control	1	1	[P1.1, P1.4, P1.3, P1.2]
17	speed (slow, fast)	1	1	[P1.1, P1.3, P1.2, P1.4]
18	speed (fast, slow)	4	1	[P1.2, P1.4, P1.3, P1.1]
19	speed (fast, fast)	4	1	[P1.3, P1.2, P1.4, P1.1]
20	speed (slow, slow)	1	1	[P1.1, P1.2, P1.3, P1.4]
21	speed (fast, fast)	2	1	[P1.4, P1.3, P1.2, P1.1]
22	speed (slow, fast)	1	1	[P1.2, P1.4, P1.1, P1.3]
23	speed (fast, fast)	1	1	[P1.1, P1.3, P1.4, P1.2]
24	speed (slow, slow)	4	1	[P1.4, P1.1, P1.3, P1.2]
25	speed (fast, slow)	1	1	[P1.2, P1.1, P1.3, P1.4]
26	speed (slow, fast)	2	1	[P1.4, P1.3, P1.2, P1.1]

Supplementary Table 1: Details about the trials carried out by Group 1. Trial condition, initial inter-personal distance (IPD) between participants, and participants' positions are specified.

**Trial**	**Condition**	**IPD** [m]	**Group**	**Pedestrian** [FL, FR, BL, BR]
1	control	2	2	[P2.4, P2.2, P2.1, P2.3]
2	speed (fast, slow)	4	2	[P2.1, P2.2, P2.4, P2.3]
3	speed (slow, slow)	1	2	[P2.2, P2.1, P2.4, P2.3]
4	speed (slow, fast)	2	2	[P2.1, P2.3, P2.2, P2.4]
5	speed (slow, fast)	4	2	[P2.2, P2.4, P2.1, P2.3]
6	speed (fast, slow)	1	2	[P2.1, P2.3, P2.4, P2.2]
7	control	1	2	[P2.4, P2.1, P2.2, P2.3]
8	speed (slow, fast)	1	2	[P2.2, P2.4, P2.1, P2.3]
9	speed (fast, fast)	2	2	[P2.3, P2.4, P2.2, P2.1]
10	speed (fast, fast)	1	2	[P2.3, P2.1, P2.4, P2.2]
11	speed (slow, slow)	4	2	[P2.4, P2.2, P2.3, P2.1]
12	speed (fast, fast)	4	2	[P2.1, P2.4, P2.2, P2.3]
13	speed (slow, slow)	2	2	[P2.2, P2.1, P2.4, P2.3]
14	control	4	2	[P2.4, P2.2, P2.1, P2.3]
15	speed (fast, slow)	2	2	[P2.2, P2.4, P2.1, P2.3]
16	direction (left, right)	4	2	[P2.1, P2.4, P2.2, P2.3]
17	direction (left, right)	2	2	[P2.1, P2.3, P2.2, P2.4]
18	direction (right, right)	4	2	[P2.1, P2.4, P2.3, P2.2]
19	direction (left, left)	4	2	[P2.2, P2.1, P2.4, P2.3]
20	direction (left, left)	1	2	[P2.4, P2.3, P2.1, P2.2]
21	direction (left, right)	1	2	[P2.3, P2.2, P2.1, P2.4]
22	direction (right, left)	4	2	[P2.3, P2.2, P2.1, P2.4]
23	direction (right, left)	2	2	[P2.2, P2.3, P2.4, P2.1]
24	direction (left, left)	2	2	[P2.1, P2.4, P2.2, P2.3]
25	direction (right, right)	1	2	[P2.1, P2.2, P2.4, P2.3]

Supplementary Table 2: Details about the trials carried out by Group 2. Trial condition, initial inter-personal distance (IPD) between participants, and participants' positions are specified.

Supplementary figures

In order to confirm our findings in the main text, in this section we show further evidence that participant P2.4 effectively assumes leadership role independent of his/her position in the group. In Figure 1, 2, 3, 4 four sample trials are visualised, where the participant is respectively on the front right, back right, front left and back left position. It is clear that participant P2.4 moves first in all conditions, acting as leader even if he/she is located in the back positions of the group. Furthermore, in the network reconstruction the same participant has only outgoing edges meaning that the participant under investigation influences the others but is seldom influenced.

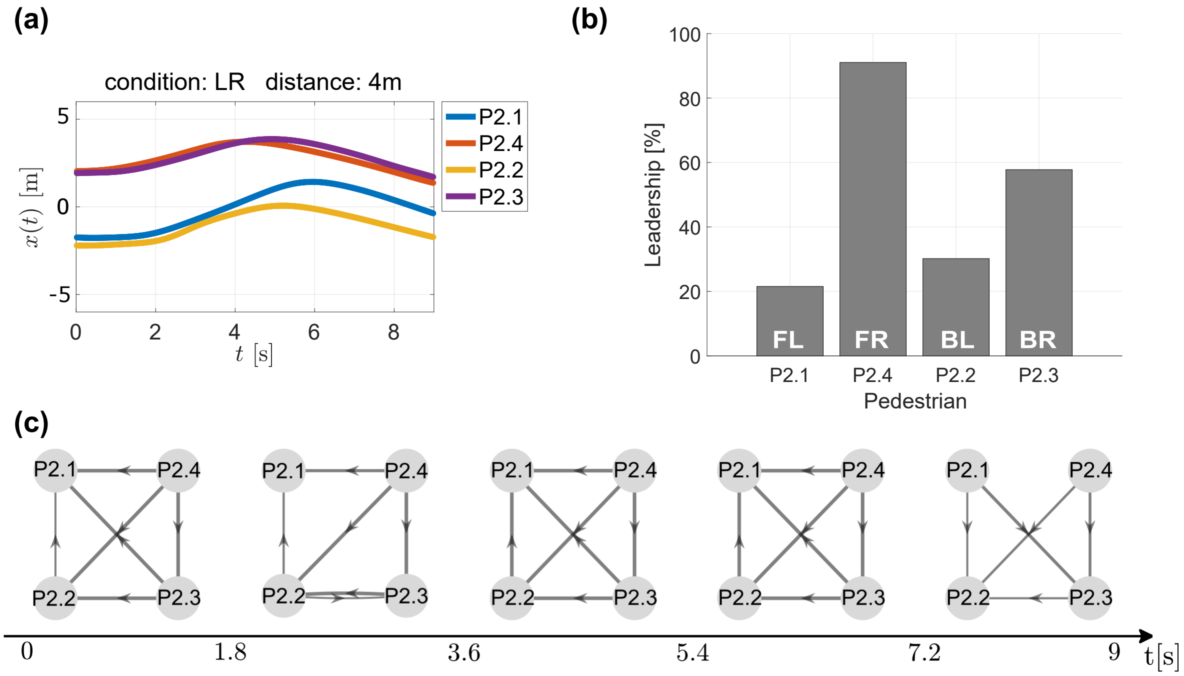

Supplementary Figure 1: **Participant P**2.4 **in the front right position.** Direction-change trial implementing the sequence "LR" (turn left, turn right) and having an initial distance between participants of 4 metres. *(a) Velocity time series.* Pedestrians' velocity time series in the *xy*-plane where the *x*-axis represents the time in seconds and the *y*-axis the horizontal component of the velocity vector. Different pedestrians are characterised by different colours and identified by the label P*x*.*y*, where *x* is the group and *y* is the specific member of the group. *(b) Percent leadership.* Each grey bar represents the percentage of leadership taken by the participant in the group during the trial. The labels FL, FR, BL, BR on each bar refer to the position occupied by the corresponding pedestrians and they are "front left, front right, back left, back right". *(c) Network reconstruction.* Each node represents a participant. The interaction between them are graphically represented through an arrow with different width, the thicker the arrow the stronger the interaction. Five different networks are reconstructed over different time-windows.

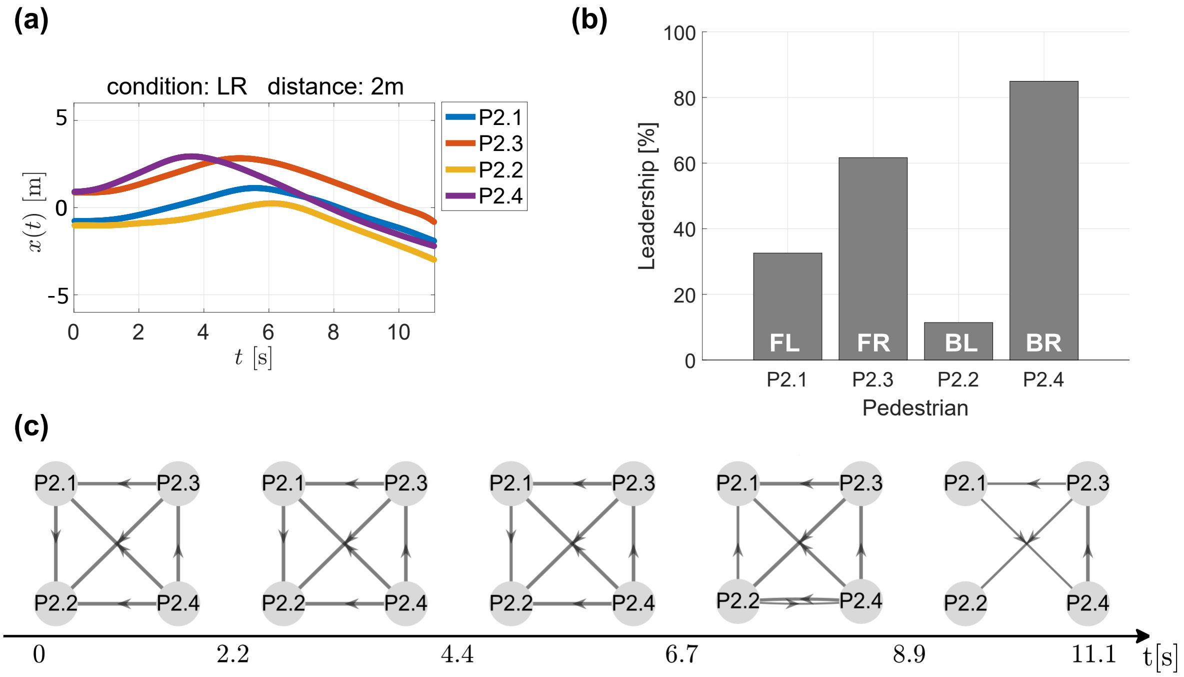

Supplementary Figure 2: **Participant P**2.4 **in the back right position.** Direction-change trial implementing the sequence "LR" (turn left, turn right) and having an initial distance between participants of 2 metres. *(a) Velocity time series*. Pedestrians' velocity time series in the xy-plane where the x-axis represents the time in seconds and the y-axis the horizontal component of the velocity vector. Different pedestrians are characterised by different colours and identified by the label P$x.y$, where x is the group and y is the specific member of the group. *(b) Percent leadership.* Each grey bar represents the percentage of leadership taken by the participant in the group during the trial. The labels FL, FR, BL, BR on each bar refer to the position occupied by the corresponding pedestrians and they are "front left, front right, back left, back right". *(c) Network reconstruction.* Each node represents a participant. The interaction between them are graphically represented through an arrow with different width, the thicker the arrow the stronger the interaction. Five different networks are reconstructed over different time-windows.

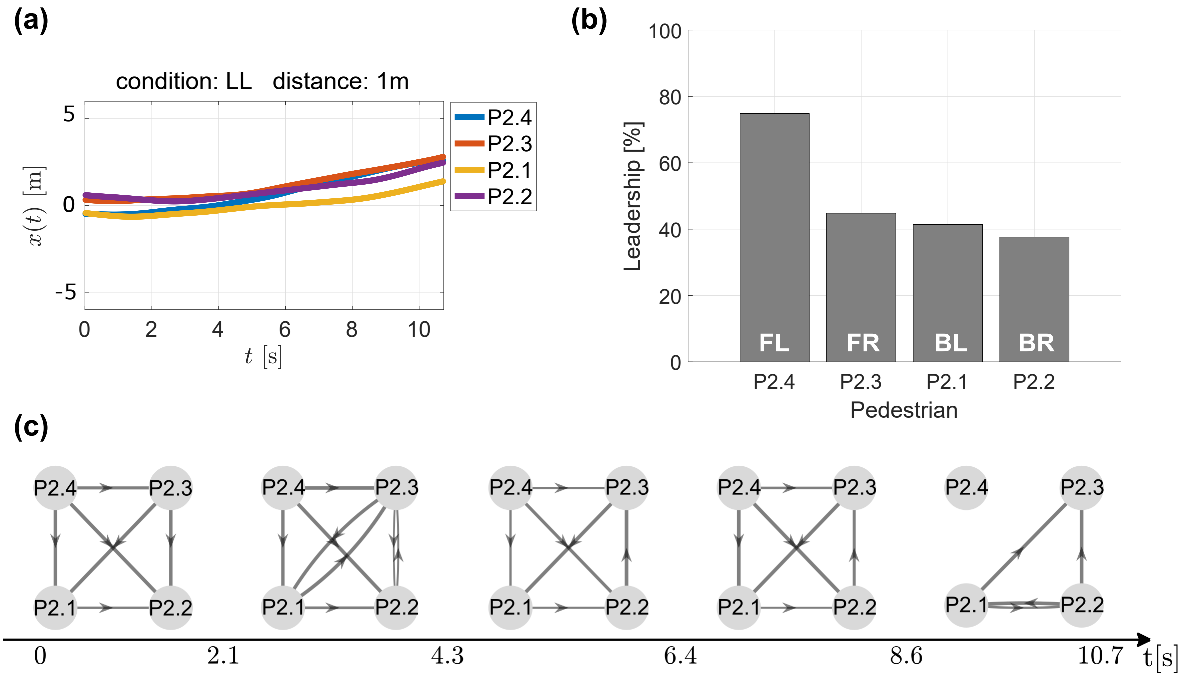

Supplementary Figure 3: **Participant P**2.4 **in the front left position.** Direction-change trial implementing the sequence "LL" (turn left, turn left) and having an initial distance between participants of 1 metre. *(a) Velocity time series.* Pedestrians' velocity time series in the *xy*-plane where the *x*-axis represents the time in seconds and the *y*-axis the horizontal component of the velocity vector. Different pedestrians are characterised by different colours and identified by the label P*x*.*y*, where *x* is the group and *y* is the specific member of the group. *(b) Percent leadership.* Each grey bar represents the percentage of leadership taken by the participant in the group during the trial. The labels FL, FR, BL, BR on each bar refer to the position occupied by the corresponding pedestrians and they are "front left, front right, back left, back right". *(c) Network reconstruction.* Each node represents a participant. The interaction between them are graphically represented through an arrow with different width, the thicker the arrow the stronger the interaction. Five different networks are reconstructed over different time-windows.

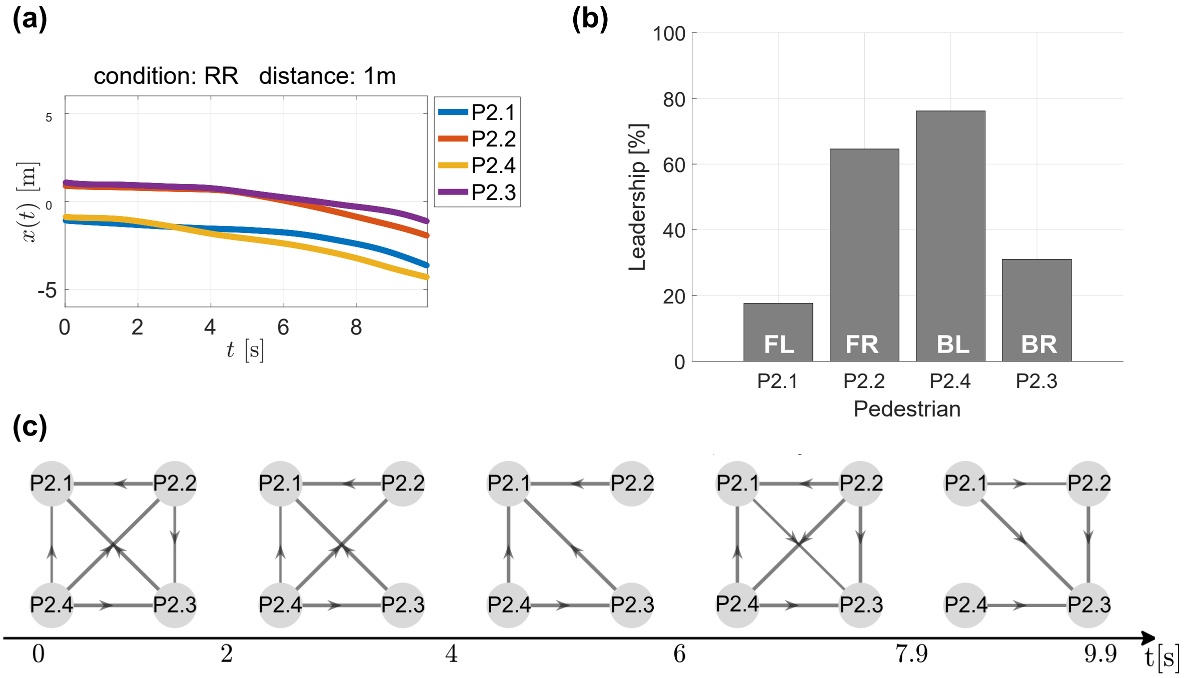

Supplementary Figure 4: **Participant P**2.4 **in the back left position.** Direction-change trial implementing the sequence "RR" (turn right, turn right) and having an initial distance between participants of 1 metre. *(a) Velocity time series*. Pedestrians' velocity time series in the *xy*-plane where the *x*-axis represents the time in seconds and the *y*-axis the horizontal component of the velocity vector. Different pedestrians are characterised by different colours and identified by the label P*x*.*y*, where *x* is the group and *y* is the specific member of the group. *(b) Percent leadership.* Each grey bar represents the percentage of leadership taken by the participant in the group during the trial. The labels FL, FR, BL, BR on each bar refer to the position occupied by the corresponding pedestrians and they are "front left, front right, back left, back right". *(c) Network reconstruction.* Each node represents a participant. The interaction between them are graphically represented through an arrow with different width, the thicker the arrow the stronger the interaction. Five different networks are reconstructed over different time-windows.
